# Eating Habits in Patients with Familial Hypercholesterolemia from North-Eastern Romania

**DOI:** 10.3390/nu14153124

**Published:** 2022-07-29

**Authors:** Alexandra Maștaleru, Alexandra Sabina Cojocariu, Andra Oancea, Maria-Magdalena Leon-Constantin, Mihai Roca, Ioana Mădălina Zota, Irina Mihaela Abdulan, Cristina Rusu, Laura Mihaela Trandafir, Alexandru Dan Costache, Elena Cojocaru, Iulia Cristina Roca, Florin Mitu

**Affiliations:** 1 Department of Medical Specialties I, “Grigore T. Popa” University of Medicine and Pharmacy, 700115 Iaşi, Romania; alexandra.mastaleru@gmail.com (A.M.); sabina.cojocariu@outlook.com (A.S.C.); andra.radulescu@yahoo.com (A.O.); roca2m@yahoo.com (M.R.); madalina.chiorescu@gmail.com (I.M.Z.); adcostache@yahoo.com (A.D.C.); mitu.florin@yahoo.com (F.M.); 2 Clinical Rehabilitation Hospital, 700661 Iaşi, Romania; 3 Department of Medical Genetics, “Grigore T. Popa” University of Medicine and Pharmacy, 700115 Iaşi, Romania; cristina.rusu@umfiasi.ro; 4 Department of Mother and Child, “Grigore T. Popa” University of Medicine and Pharmacy, 700115 Iasi, Romania; laura.trandafir@umfiasi.ro; 5 Department of Morphofunctional Sciences I, “Grigore T. Popa” University of Medicine and Pharmacy, 700115 Iaşi, Romania; elena2.cojocaru@umfiasi.ro; 6 Surgery II Department, “Grigore T. Popa” University of Medicine and Pharmacy, 700115 Iaşi, Romania; iuliaroca@yahoo.com

**Keywords:** familial hypercholesterolemia, food frequency questionnaire, eating habits

## Abstract

(1) Background: Familial hypercholesterolemia (FH) is a genetic autosomal dominant disorder characterized by elevated levels of low-density lipoprotein cholesterol (LDL) that develops deposits of lipids in the arterial wall. Since it is underdiagnosed and undertreated, the disease has a high risk of premature cardiovascular disease and death. Patients are not always aware of the changes they should make in their diet. Thus, our study aimed to evaluate through a food frequency questionnaire their eating habits. (2) Methods: We included 70 patients with FH and 20 subjects in a control group that were evaluated through a physical examination and blood tests. They also completed a food frequency questionnaire. (3) Results: Throughout our study, we observed several aspects: regardless of age, patients with FH had higher carbohydrate intakes compared to the control group; from the same group, a positive correlation was observed between salami intake and the levels of glucose and glycated hemoglobin. Moreover, the sour cream preference was associated with higher liver function tests. In the control group, we observed a higher intake of pasta and fast food and fewer fruit portions. (4) Conclusions: As far as we know, this is the first study from Romania that evaluated the eating habits of patients diagnosed with FH. Our study reveals that, although patients with FH avoid junk food, they still have a high intake of carbohydrates when compared to the control group. Further research is needed in order to get a comprehensive nutritional evaluation of these patients.

## 1. Introduction

Familial hypercholesterolemia (FH) is a genetic autosomal dominant disorder characterized by elevated levels of low-density lipoprotein cholesterol (LDL) that develops deposits of lipids in the arterial wall, thus leading to a high risk of premature cardiovascular disease [1]. The reported prevalence of FH varies between countries and continents. A recently published meta-analysis that included more than 80% of the cases from the European general population, evaluated the prevalence as being 1:311, ranging between 1:200 and 1:575 [2]. The subjects with FH generally have a mutation in one of the following genes: the low-density lipoprotein receptor gene (LDLR—the most common), the apolipoprotein B gene (APOB3500), and a gain-of-function type of mutation of the proprotein convertase subtilisin kexin 9 gene (PCSK9). Any of those mutations inhibit the catabolism of LDL receptors, resulting in elevated LDL serum concentrations [3].

It is well-known and described by the latest European Society of Cardiology guidelines that patients diagnosed with FH are at very high cardiovascular risk. Thus, they need rigorous medical treatment in order to achieve the LDL target of less than 55 mg/dL [4] but also an important change in their daily routine regarding physical activity and dietary habits [5].

The nutritional assessment of patients with FH is a complex one and includes objective methods (clinical examination, anthropometric measurements, and laboratory tests) but also subjective ones. The last category includes a food diary, the daily nutritional intake, and the food frequency questionnaire. The first two require extra effort on the part of the patients, as they have to weigh the food and write down the details of each meal in a diary. The food frequency questionnaire provides information on the regularity of different food groups’ consumption [6,7].

Studies performed on patients with FH are very few, being almost nonexistent when it comes to correlating the biological parameters with nutritional assessments. Moreover, knowing their dietary patterns regarding their favorite foods, the types of fat used for cooking, the weekly frequency of different classes of aliments, and their feelings after overeating will provide us with a better understanding of their nutritional needs. Being the first of its kind, this study aimed to obtain data on the eating habits of patients with FH but also to compare them to a control group. Such research would make possible future nutritional interventions as a complementary measure in the management of such complex patients.

## 2. Materials and Methods

### 2.1. Study Design and Setting

This cross-sectional study was conducted in the Cardiovascular Rehabilitation Clinic from Iasi Clinical Rehabilitation Hospital (Romania) between 1 December 2020 and 31 March 2022. Our study included patients with familial hypercholesterolemia, a diagnosis that was sustained based on a Dutch Lipid Clinic Network (DLCN) score of more than 8 points. If the DLCN score was greater than 8, it was classified as a definite FH case, probable if the patient accumulated between 6 and 8 points, possible between 3 and 5 points, and unlikely to have FH below 3 points. Due to the fact that most of the patients already had in their daily administration a lipid-lowering treatment (LLT), we adjusted the values used in the DLCN score with a correction factor. For example, in a patient treated with 10 mg of Atorvastatin, the LDL value was corrected by multiplying the actual result by 1.6, while for the ones treated with 20 mg, the correction factor was 1.8 [8]. Therefore, untreated LDL was considered if the patient had six or more months of the absence of any type of LLT; thus, the LDL value included in our study was the one validated by our laboratory. On the other hand, pretreated LDL values were corrected in order to observe the impact that the LLT had on the lipid profile values. The correction factor used is different for each type and dose of statin administered, alone or in association with ezetimibe.

### 2.2. Study Participants

**Figure 1:**
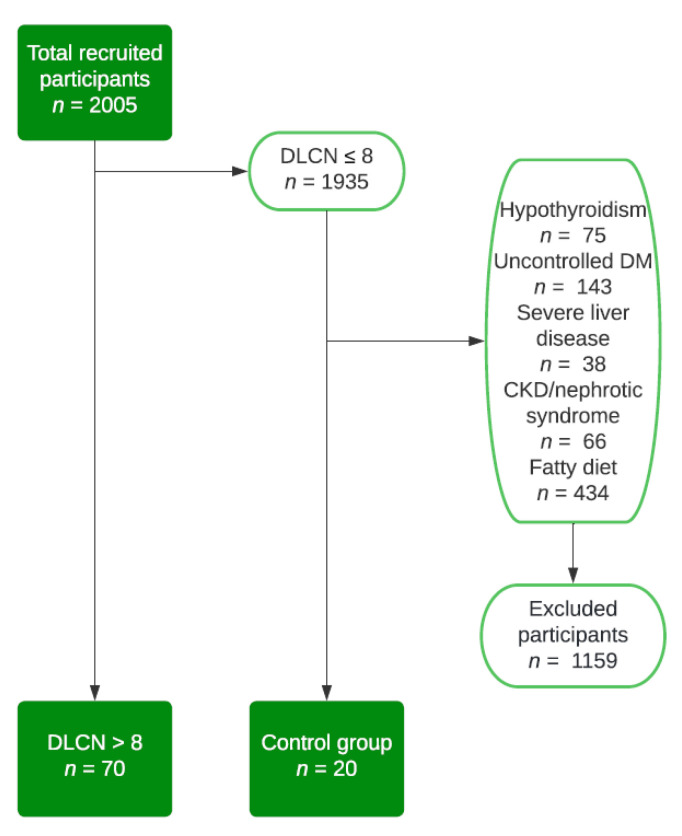
Flow chart—study group selection. DLCN, Dutch Lipid Clinic Network; DM, diabetes mellitus; CKD, chronic kidney disease.

Between 1 December 2020 and 31 March 2022, we identified, from 2005 patients that were admitted to our clinic, 70 patients with FH that satisfied the inclusion criteria in order to take part in our study. All our patients were older than 18 years old, signed the informed consent, and had more than 8 points in the DLCN score. In addition, we included 20 patients in the control group that had a DLCN score ≤ 8 points with a normal lipid profile. Patients who did not fulfill these criteria were not taken into consideration in the research. In order to rule out the possibility of secondary hypercholesterolemia, patients who presented at least one of the following conditions were excluded from the study: diabetes mellitus with uncontrolled glycemic values, hypothyroidism, nephrotic syndrome, severe chronic kidney disease, severe liver disease, and meals with higher saturated and trans-unsaturated fatty acids (Figure 1).

### 2.3. FH Patients Evaluation

A single investigator carried out both the anamnesis and the physical examination and collected the following information: sex; age; education (gymnasium, high school, university, and 10 classes or less); employment status (working full-time, part-time, retired, or not working); and the body mass index.

The following types of blood tests were carried out on the patients who were enrolled: hemoglobin (Hb, md/dL), uric acid (UA, mg/dL), iron (µg/dL), urea (mg/dL), creatinine (mg/dL), proteins (g/dL), aspartate transaminase/serum glutamic oxaloacetic transaminase (AST/GOT, UI/L), alanine aminotransferase/serum glutamic pyruvic transaminase (ALT/GPT, UI/L), gamma-glutamyl transpeptidase (GGT, U/L), blood glucose (mg/dL), glycated hemoglobin (HbA1c, %), total cholesterol (TC, mg/dL), triglycerides (TG, mg/dL), high-density lipoprotein (HDL, mg/dL), and LDL (mg/dL). These laboratory tests were performed using an Erba XL 1000 Spectrophotometer^®^ (Erba Diagnostics, Mannheim, Germany).

The same investigator applied the food frequency questionnaire to all the patients included in this research. The questionnaire includes the number of meals eaten per day, number of snacks, favorite food, types of fats used for cooking or consumed, the frequency of the eating habits, what makes the patient eat more, and what feelings the patient has after overeating. 

### 2.4. Ethical Approval

In order to be enrolled in the research, all of the patients had to complete a written informed consent form. Our study received approval from the Ethics Committee of both “Grigore T. Popa” University of Medicine and Pharmacy Iasi (certificate of approval dated 15 June 2020) and Iasi Clinical Rehabilitation Hospital (certificate of approval dated 25 November 2020).

### 2.5. Statistical Methods

Data analysis was performed using SPSS 20.0 (Statistical Package for the Social Sciences, Chicago, IL, USA). For continuous data, the normality of the distribution was assessed by the Shapiro–Wilk test. Data were expressed as the mean ± standard deviation, as a median with an interquartile range (IQR), or a number of cases with a percent frequency for continuous variables with normal distribution, for non-normally distributed continuous variables, and for categorical variables, respectively. Continuous variables with normal distribution were compared using independent samples for the Student’s *t*-test in the case of two samples or applying one-way ANOVA in the case of three or more independent groups. The comparisons for continuous variables not satisfying the assumption of normality were done by applying nonparametric tests: the Mann–Whitney *U* test in the case of two samples or the Kruskal–Wallis test for more than two samples. Comparisons of the categorical variables were done by the chi-square test or by Fischer’s exact test when the expected values in any of the cells of a contingency table were below 5. A correlation analysis was done using the Spearman’s correlation test, considering that this analysis involved only non-normally distributed variables. A two-sided *p*-value <0.05 was considered significant for all statistical analyses.

## 3. Results

Our research included a study group consisting of 70 patients diagnosed with FH and 20 patients with a normal lipid profile as the control group. All the general characteristics can be observed in Table 1.

After applying the Dutch Lipid Clinic Network diagnostic criteria, we observed that 47.1% had a first-degree relative with premature cardiovascular disease, and 30% had a first-degree relative with a LDL value over the 95th percentile. More than 35% of the included patients were diagnosed with cardiovascular disease, and almost half of the study group had arcus cornealis before 45 years. Forty precent of the patients had an LDL value above 330 mg/dL, accumulating 8 points in the Dutch Lipid Clinic Network diagnostic criteria. The criteria used for the diagnosis of the patients are described in Table 2. 

In Table 3, we can observe the eating habits of patients from the two studied groups. 

A statistically significant difference can be observed between the two studied groups regarding the number of subjects that preferred eating sour cream (*p* = 0.007). We also noticed differences between the means/medians in the FH group as follows: the habit of eating meat was associated with higher hemoglobin values and the preference for salami with elevated values of hemoglobin, glucose, glycated hemoglobin, GPT, and the body mass index (BMI), while fruits increase HDL cholesterol. Moreover, the patients with a food preference for bread had higher BMI and GGT values, while the subjects who chose sour cream as a favorite food had higher liver enzymes. The patients that had potatoes as a favorite aliment had higher BMIs but also elevated values of glycated hemoglobin and GGT values, while the subjects who were more into sweets had higher glucose values. In addition, fast food was strongly associated with higher GTP values, and the habit of eating cakes was negatively associated with the creatinine value (Table S1 from the Supplementary Materials). 

On the other hand, in the control group, we observed that vegetable consumption was associated with higher LDL cholesterol and hemoglobin concentrations. In addition, the patients that had cheese as their favorite food had higher values of hemoglobin, proteins, and creatinine clearance, while dairy product consumers had higher iron and GPT concentrations. Negative associations were observed between the subjects that had cakes as their preferred food and their GGT values and between juice drinkers and their triglyceride values (Table S1 from the Supplementary Materials).

We have also evaluated the eating habits of the patients with FH divided into age groups. The patients included in our study between 18 and 24 years old did not have preferred food aliments such as bread, cheese, or sweets, all of them being preferred by at least 40% in almost all the other group ages. Vegetables and dairy products were preferred by all the age categories, the smallest percentage being observed in the 25–34 years old group. These results can be observed in Table S2 from the Supplementary Materials.

Table 4 describes the type of fats used for cooking or frequently consumed in both studied groups. 

Statistical significance was obtained for the patients included in the FH group between the hemoglobin value and their preference in using bacon for cooking or consuming it. Additionally, we observed an association between bacon consumption and higher values of GGT, GPT, glucose, glycated hemoglobin, uric acid, and triglyceride values. Moreover, a higher butter consumption was associated with lower iron values. In addition, we detected a negative correlation between butter consumption and the GGT, urea, glucose, and glycated hemoglobin values. Furthermore, frequently using sour cream was associated with elevated levels of hepatic enzymes: GGT, GOT, and GPT but also with the proteins and the uric acid value. Additionally, a statistically significant difference was observed between cooking with oil and elevated creatinine, uric acid, and triglycerides values and between margarine consumption and higher protein values (Table S3 from the Supplementary Materials).

Moreover, we assessed the types of fats used by the age groups. While the patients included in the group 18–24 years old ate only butter and vegetable oil, all the individuals included in our study aged over 45 also consumed lard and margarine. Sour cream was preferred by patients between 25 and 74 years old, while whipped cream was preferred by individuals between 35 and 74 years old. The results can be observed in Table S4 from the Supplementary Materials.

Table 5 describes the frequency of the usual eating types of food in both studied groups. 

In our study, we noticed in the FH group that a higher vegetables and beef/pork meat intake was associated with elevated urea values. Moreover, a negative association was observed between fish consumption and GOT, GPT, total cholesterol, and non-HDL concentrations (Table S5 from the Supplementary Materials).

In addition, we evaluated the frequency of the eating habits in the age groups. When considering bread, the patients with FH included in our study aged 18–24 years old ate bread only two to three times/week, while the elderly ones ate bread mostly more than four times/week. Regarding the vegetables, potatoes were eaten by most of the patients older than 35 years a maximum of four to six times/week, while green peas were often consumed a maximum of two to three times/week. When evaluating the meat consumption, beef/pork was usually eaten less than six times/week, while chicken was consumed even daily in patients between 35 and 64 years old. Fried food was eaten by all ages mostly up to four to six times/week, while 4–9% of the patients between 45 and 74 years old consumed it daily. These results can be observed in Table S6 from the Supplementary Materials.

Table 6 describes the situations in which the patients with FH and the control included in our study ate more than they should. 

As seen in Table 5, in order to facilitate the reading of the text, we used the following abbreviations regarding the frequency of the weekly consumption: 1—less than two times/week, 2—two to three times/week, 3—four to six times/week, and 4—daily. The rejection of the null hypothesis was observed when we compared how often they ate fried food and the feeling of loneliness (3(2 to 3) vs. 2(1–3), *p* = 0.038) and during the weekend (2(2 to 3) vs. 2(1 to 2), *p* = 0.018). The same test revealed that the patients with FH ate more pasta due to depression (2(1.75 to 2) vs. 1(1 to 2), *p* = 0.016), potatoes due to stress (2(2 to 3) vs. 2(1 to 2), *p* = 0.028), and less fruits in front of the television (3(2 to 3) vs. 3.5(3 to 4), *p* = 0.035). Moreover, the patients diagnosed with FH ate more potatoes (2(2 to 3) vs. 2(1 to 2), *p* = 0.016), green peas (2(1 to 2) vs. 1(1 to 2), *p* = 0.007), and other vegetables (4(3 to 4) vs. 3(2 to 3), *p* = 0.007) in society.

On the other hand, in the control group, we observed the rejection of the null hypothesis in the subjects that ate fewer vegetables due to boredom (1.5(1 to 2.75) vs. 3(3 to 3), *p* = 0.029).

Further, we evaluated these situations divided into age groups for the patients with FH. While people included in the 18–24 years old group ate more due to stress and on the weekend, people aged more than 45 years old ate more due to loneliness, sadness, depression, or stress but also in society, on the weekend, or in front of the television. None of the patients included in the 18–24 years old or more than 75 years old groups ate more due to boredom. All these results can be observed in Table S7 from the Supplementary Materials.

In Table 7, we examined the responses of our patients with FH and from the control group regarding their feelings after they ate more. 

After applying the Mann–Whitney test, we observed that the patients with FH felt depression when eating too little cheese (1(1 to 2) vs. 2(2 to 3), *p* = 0.038); guilt after eating fast food (1(1 to 2) vs. 1(1 to 1), *p* = 0.005); and satisfaction after eating pasta (2(1.5 to 2) vs. 1(1 to 2), *p* = 0.015) and green peas (2(1.5 to 2) vs. 1(1 to 2), *p* = 0.033). Moreover, the patients who ate beef/pork did not undergo any physical activity (2(1 to 2) vs. 2(2 to 3), *p* = 0.018).

Furthermore, we evaluated the responses given by the patients with FH on the age groups. In the case of overeating, we noticed that the patients aged between 18 and 24 years old did not experience any feelings, while 27–50% of the patients between 35 and 74 years old exercised. The old people included in the group of more than 75 years either did nothing or skipped the next meal. These results can be observed in Table S8 from the Supplementary Materials.

### 3.1. Patients ≥65 Years Old

In order to gain a better understanding of the patients included in our study, we evaluated their frailty status and their degree of malnutrition by applying the MNA questionnaire.

Some (18.8%) of the patients over 65 years old had a risk of malnutrition, all of them being females. Regarding the frailty status, 6.3% were not frail, 6.3% were prefrail, and 87.5% of the patients more than 65 years old were frail. 

When declaring the number of meals and snacks eaten per day, 75% of the elderly patients had three meals, and half of them had two snacks per day. Regarding breakfast, 68.8% always ate it, 25% ate it sometimes, and 6.3% never ate it in the morning. When referring to the food preferred by the elderly, many ate bread (68.8%), vegetables (75%), cheese (87.5%), dairy products (75%), and meat (68.8%), while most of them did not eat salami (68.8%), sweets (75%), juices (87.5%), or cakes (62.5%).

Moreover, we observed after applying the *t*-test that there was a statistically significant difference when comparing the Mini Nutritional Assessment (MNA) score with the response ‘depression’ in patients who ate more than they should (*p* = 0.018). In addition, a statistically significant difference was also observed between the MNA score and frailty (*p* = 0.038). 

### 3.2. Educational Status

When evaluating the patients with FH that finished gymnasium, we observed that 85.7% preferred meat, fruits, and potatoes; 71.4% had as their favorite food bread, vegetables, and cakes; 57.1% preferred sour cream; 42.9% chose sweets; 14.3% soft juices and bacon; and all of them ate dairy products. A majority (71.4%) of the patients with FH from this category ate bacon, in contrast with only 15.9% of the subjects who had higher education and a lower intake (*p* = 0.004). Moreover, 42.9% of the same subjects ate more due to depression, compared to 11.1% of the patients that were depressed but finished a higher type of education (*p* = 0.023). From the control group, only 50% of the subjects that finished gymnasium used oil for cooking, while all the patients with higher education used it for cooking (*p* = 0.002). 

In patients with FH that graduated from 10 classes, we observed the rejection of the null hypothesis, explained by eating more fruits (4(3 to 4) vs. 3(2–4), *p* = 0.027) and less sweets (2(1 to 2) vs. 2(1–3), *p* = 0.026).

The subjects with FH that finished high school had different scores when referring to their favorite food: 87% ate fruits, 82.6% liked vegetables, 78.3% preferred potatoes, 69.6% ate meat and dairy products, 65.2% chose bread, 60.9% had sour cream as a favorite condiment, and 52.2% selected bacon from the list. Some (43.5%) had as their preferred food sweets, 26.1% preferred cakes, and only 13% chose to drink soft juices. A majority (60.9%) of the patients with FH that used sour cream for cooking finished high school, compared to only 34% from the other educational categories (*p* = 0.042). In addition, 34.8% of the patients included in this group ate more due to loneliness, compared to only 12.8% from the other educational status groups (*p* = 0.031). In the control group, all the subjects that finished high school had as a favorite food salami (vs. 16.7%, *p* = 0.010) or fast food (vs. 27.8%, *p* = 0.042) and ate more due to boredom (vs. 11.1%, *p* = 0.032) when compared to the subjects with other educational statuses.

Moreover, among the FH patients that graduated university studies, 86.2% chose meat, 82.8% ate vegetables, 65.5% liked fruits and cakes, 62.1% ate dairy products, and 58.6% preferred bread. A lot of them (55.2%) ate sweets, 51.7% liked potatoes, and 34.5% had bacon as their favorite food. Finally, 27.6% ate sour cream and fast food, and 13.8% drink soft juices. Regarding the patients that finished university studies, 65.5% of them had cakes as their favorite food vs. 39% of the patients with a lower educational status (*p* = 0.029). Moreover, only 27.6% used sour cream for cooking, while 58.6% of the patients with a lower educational degree ate it (*p* = 0.049). In addition, 51.7% of the patients had as their favorite food potatoes, compared to 75.6% from the other educational categories (*p* = 0.045). When taking into consideration the situations that made the subjects from this group eat more, 58.6% admitted that they ate more during the weekend, 55.2% due to stress, and 24.1% noted boredom compared to 29.3%, 19.5%, and 53.7% from the other educational categories (*p* = 0.026; *p* = 0.004; *p* = 0.016). Additionally, the same subjects responded that, after a fulfilling meal, 41.4% skipped the next meal, compared to only 14.6% from the other educational categories (*p* = 0.025). In the control group, all the subjects that had a lower educational status had as their favorite food meat (vs. 46.7%, *p* = 0.035), dairy products (vs. 40%, *p* = 0.038), and potatoes (vs. 40%, *p* = 0.038) when compared to the subjects that finished university studies. Moreover, only 13.3% of the subjects with high education preferred salami, while 60% of the individuals with other educational statuses ate it (*p* = 0.037).

### 3.3. Employment Status

When considering the patients with FH that worked part-time, 66.7% of them were depressed after overeating, unlike people with other forms of employment, where the percentage was only 7.5% (*p* = 0.025). Additionally, in the part-time category, the patients ate less chicken (1(1 to 1) vs. 2(13), *p* = 0.025), beef/pork meat (1(1 to 1) vs. 2(1–3), *p* = 0.023), and fried foods (1(1 to 1) vs. 2(1–3), *p* = 0.029).

The patients with FH that worked full-time declared a higher consumption of chicken (2(2 to 3) vs. 1.5(1 to 2), *p* ≤ 0.001). Regarding the patients from the control group that worked full-time, only 43.8% of them had potatoes as their favorite food, while all the patients with other employment statuses preferred them (*p* = 0.043).

Finally, 66.7% of the patients with FH that were not employed ate higher amounts of lard when compared to the employed or retired patients, where the percentage was 13.4% (*p* = 0.013). Similar findings were also observed for margarine (*p* = 0.029). We also noticed a rejection of the null hypothesis, where the retired patients ate more meals (3(2 to 3) vs. 2 (2 to 3), *p* = 0.021), rarely ate breakfast (1(1 to 2) vs. 2(1 to 2), *p* = 0.036), and consumed chicken occasionally (1.5(1 to 2) vs. 2(2 to 3), *p* = 0.002).

### 3.4. Civil Status

Among the favorite foods in the married FH patients, we observed vegetables (85.7%), meat (80.4%), and fruits (78.6%). About half of them preferred bread and sweets. The most commonly used fat in cooking was oil (92.9%), followed by sour cream and butter (51.8%, 50%). Moreover, 82.1% of the married patients with FH had cheese as their favorite food, while only 50% of the patients that were not married had this food habit (*p* = 0.031). In addition, 37.5% of the married subjects felt guilt after overeating, compared to only 7.1% of the patients with other marital statuses (*p* = 0.029). From the control group, 75% of the married subjects had bread and sweets as their favorite foods, while only 25% of the individuals with other civil statuses had these preferences (*p* = 0.028).

We noticed in our study a rejection of the null hypothesis; thus, when comparing the number of divorced patients with FH and the frequency of eating other vegetables, we observed that they ate less vegetables compared to others (1(1–3) vs. 3 (2–4), *p* = 0.033).

Regarding the widowed patients with FH, only 42.9% had cheese as their favorite food, while 79.4% of the subjects with other marital statuses had the same food preference (*p* = 0.033). Similar findings were observed regarding dairy products: while 28.6% of the widowed patients had them as their favorite food, 71.4% of the patients that had other civil statuses also favored them (*p* = 0.035). We observed through the rejection of the null hypothesis that widowers ate fewer sweets compared to those with other marital statuses (1(1 to 2) vs. 2(1–3), *p* = 0.034). In addition, 57.1% of the widowed patients ate more in front of the television compared to 22.2% of the subjects with other civil statuses (*p* = 0.045).

We also observed the rejection of the null hypothesis between the number of patients with FH that had never been married and the frequency in bread consumption, these subjects eating less bread when compared to the other civil statuses (2(2 to 2) vs. 4(3 to 4), *p* = 0.046).

## 4. Discussion

Analyzing the favorite foods of the studied group, we noticed the preference for cheese, meat, and vegetables. On the other hand, juices were the least favorite food on the list. Potatoes were a staple food, most probably due to their low price and the high number of potato cultures in the whole Romanian territory.

Although there were no statistically significant differences, it is worth mentioning that, in the FH group, we noticed a clear preference for sour cream, cakes, and meat, while those in the control group preferred a higher percentage of fast food, juices, and sweets. A possible explanation may be the lower average age in the control group. However, comparing the young patients from the two groups, we observed that those from the FH group lacked in eating bread, cheese, sour cream, sweets, and juices. We believe that these findings were present due to the fact that young people were more rigorous in their lifestyle changes when they were diagnosed with FH compared to the other age groups or with those with no health issues. The results of another study from Norway showed that the food choices of patients with FH are much more prudent than those of people with lifestyle-induced hypercholesterolemia, most likely because of the awareness of the disease and increased motivation for a healthy lifestyle [9].

A study from 2013 about the eating habits of children and young adults (12–28 years old) with hypercholesterolemia vs. those without this disease mentioned that young people from the FH group had a healthier diet. However, the intake of sweets (juices, jam, and honey) was significantly higher, suggesting that their food education focused mainly on healthy fats and less on limiting sweets [10]. 

Similar data on young people was also observed in our study: sweets were preferred by all subjects from the 25–34 years old group, decreasing progressively with aging. On the other hand, cakes were preferred by more than 30%, regardless of age. Half of our study group ate sweets a maximum of three times a week. Furthermore, the 25–34 years old group went beyond the parameters, consuming them almost daily. In terms of juices, the results were also consistent, most of the patients from both groups consumed them mostly at a maximum of two times a week. Thus, the patients diagnosed with FH ate more food with a high glycemic index, most likely because they tried to avoid food with high cholesterol, replacing it with sweets. For the control group, we observed that a higher cake intake was associated with lower GGT values. Nanri et al. evaluated the dietary patterns and GGT values in a 9803 Japanese cohort, and they observed a negative association between the GGT values and desserts, results that were consistent with ours [11]. 

Lard, margarine, and bacon were consumed by a small number of people, which can be justified by the nature of the batch, most of the participants being from urban areas. About 60% of the group ate fried foods up to three times a week. Worrying is the fact that one-third of the group ended up eating it four to six times/week. Bacon is widely eaten and preferred due to its taste and texture but also because of its high-fat and low-lean composition [12]. The pork meat is highly loaded in saturated fatty acids; thus, a constant, increased ingestion will lead to a higher cardiovascular risk [13]. In our study, we observed a positive relationship between bacon consumption and glucose, glycated hemoglobin, and triglyceride values. Zelber-Sagi et al. demonstrated in a cohort of patients from Israel that a high consumption of red meat full of saturated fat will determine both non-alcoholic fatty liver disease and insulin resistance [14]. Moreover, Zhu et al. evaluated in rats that ate pork fat the effects on their lipid profiles. The authors observed significantly higher values of triglycerides when compared to the control, results that were in accordance with the present study [15]. 

We notice a higher consumption of carbohydrates, regardless of their type. Bread consumption was present daily in both groups, with a higher frequency in the HF group. Pasta was reported with a maximum frequency of two to three times a week, more prevalent in the control group. New studies recommend that people with hypercholesterolemia limit their carbohydrate intake. For example, Gjuladin-Hellon et al. published in a meta-analysis and systematic review on low-carbohydrate diets and cardiovascular risk that carbohydrate restrictions for a minimum of six months appear superior in improving the lipid levels when compared to low-fat diets [16]. The results seem strong enough to initiate similar studies in patients with FH, especially in those with increased insulin resistance.

Regarding the preference for vegetables, both the FH group and the control group had similar percentages. Despite that most of the patients included in both of our study groups considered potatoes as a favorite food, they ate it a maximum of three times/week. Green peas were consumed less than two times/week by more than half of the FH subjects, regardless of age, and more than 80% of the subjects from the control group. Other vegetables were eaten more than four times/week by more than 50% of the patients with FH older than 35 years old. For the FH group, we noted a correlation between the preference in eating vegetables and beef/pork meat and higher urea concentrations. In accordance with our findings, Cases et al. published an article that evaluated the diets based on vegetables and described the urea levels decreasing in patients that followed fiber-rich diets [17]. A possible explanation for these results could be the association of meat with vegetables, thus increasing the urea concentration. On the other hand, for the control group, vegetable consumption was associated with the hemoglobin value. Similar findings were published by Ghose and Yaya, who evaluated vegetables and fruit consumption and their association with anemia. Thus, the women from urban areas had a higher prevalence of developing moderate-to-severe anemia if they consumed less than five servings of vegetables and fruits [18].

Another important finding was the lack of fruits in the diet. Most patients did not consume them every day. However, the rate was higher in the FH group. Kurowska et al. published a study in which they studied the effect of orange juice on the HDL value in hypercholesterolemic patients. The subjects drank orange juice in different quantities for a period of four weeks. After this period, the authors observed an increase in the HDL value by 21% when consuming 750 mL of fresh orange juice [19]. Similar findings were also observed in our study, *p* < 0.05 being observed between fruit consumption and the HDL value.

Dairy products are also deficient in daily meals. About 70% of those with FH aged 45–64 consumed them less than three times a week. Similar findings could be observed in the control group, where 65% of the subjects ate them a maximum of three times a week. In our study, we detected a positive correlation between the preference for sour cream consumption and liver function tests. A recently published study by Watzinger et al. evaluated the dietary factors that affect the liver fat content. They observed that a higher intake of high-fat dairy, such as sour cream in our study, is associated with non-alcoholic fatty liver disease, thus increasing the liver function tests, results that were in accordance with ours [20].

Meat is another part of the diet. Beef/pork was cooked by patients with FH mostly up to three times a week for all the group ages. In the control group, only 20% of them ate it more than four times a week. When it comes to chicken meat, patients aged over 75 years with FH ate it no more than twice a week, while two-thirds of the 25–34 years old group consumed it four to six times, the rest preferring it less than three times a week. An explanation for this result could be the low price of chicken; it cooks quickly and is versatile. Fish appeared a maximum of two times on their weekly menu for most of the patients, a possible explanation being the high cost compared to other types of meat. We observed in our study a negative correlation between the pattern of eating fish and the total cholesterol value for the patients diagnosed with FH. Similar findings were described by Bulliyya, who evaluated the differences in the lipid profile between the people that ate fish and the non-fish-eating subjects. More precisely, the mean value of the total cholesterol was lower in the people who ate fish, compared to the others [21]. 

In the FH group, we observed a positive correlation between the preference for salami and glucose and, respectively, glycated hemoglobin values. There are multiple methods through which the meat is processed, such as drying, curing, or smoking. In numerous cuisines, the meat is usually maturated and fermented. The last process is based on the chemical reactions of the added sugars [22]. Thus, multiple studies have demonstrated a positive correlation between processed meat intake and high values of fasting glucose. Fretts et al. observed that, for every additional 50 g of processed meat per day, an increase of 0.021 mmol/L in the fasting glucose was present [23]. A metanalysis published in 2017 also showed a positive association between the risk of developing diabetes and an amount of 50 g of processed meat added to the daily meals [24].

When taking into account the hemoglobin value in the FH group, we observed its association with meat, salami, and bacon consumption. The strong relationship between these parameters is well-known. Tong et al. described in a recently published article that the subjects that do not eat red meat or eat a small amount have lower hemoglobin concentrations and had a higher risk of developing anemia [25]. 

Fast food dishes appeared with a limited frequency in the daily diet, most of our participants consuming them up to two times a week. For the FH group, we observed a statistically significant correlation between fast food consumption and GPT value. Kechagias et al. evaluated healthy subjects that had to eat at least two fast food meals per day and compared them with a control group. The authors objectified significantly higher values of the GTP in subjects that ate fast food compared to the controls, results that were in accordance with ours [26].

Overeating was triggered by boredom and was more frequent on the weekends in the FH group. None of the control patients felt loneliness or depression, one possible explanation being the lower mean age. Another aspect mentioned is the fact that about half of the control group ate excessively during stressful periods, on the weekends, or in society. Emotional eating is a trigger for eating imbalances and obesity. This phenomenon occurs in an attempt to cope with negative feelings or situations, in order to obtain distraction or comfort [27]. A systematic review and meta-analysis published in The American Journal of Clinical Nutrition that included thirteen observational studies noted that people with a healthy diet had a lower risk of developing depression, while a relationship was observed between depression and the Western diet [28]. Similar results were published recently by Park, Kim, and Lee in research that included 3388 middle-aged Koreans. The authors described a statistically significant association between an unhealthy dietary pattern and depression [29]. 

Another aspect that is worth mentioning is that the traditional perspective on the diets of people with FH lacks in solid evidence for the recommendations made so far. In addition, a fat-restricted diet can lead people with FH to consume excess carbohydrates, which is obviously counterproductive, as this diet can increase insulin resistance [30].

### 4.1. Patients ≥65 Years Old

Geriatric patients have different eating habits from younger adults, as the protein-caloric requirement is modified. In addition, they are conditioned by chronic diseases that bring with them restrictive diets low in salt, sugars, or fats [31]. Not least, economic, psychosocial, and physiological factors that appear with aging can be obstacles to a healthy, balanced diet.

In our group of FH, their nutritional status was normal, with only 18.8% of patients being at risk of malnutrition. Three-quarters of them had three meals a day, and their favorite foods were cheese, vegetables, and meat. Breakfast is almost always present in the habits of elderly patients, a fact also noted in other studies [32]. We observed the elimination of processed meat products, sweets, and juices from their daily diets. Previous studies showed the influence of educational level and living environment: older people in urban areas with higher education probably have a healthier diet through better access to information and quality products [33]. 

The presence of frailty in 87.5% of participants over the age of 65 may explain the choice of foods that are easier to prepare and integrate into the daily diet: meat, vegetables, and cheese.

According to a series of studies in the Balkans, fish is not among the favorite foods, possibly due to the high price, but also because it is not found in their traditional cuisine. Thus, we can explain the percentages found in our research: 37.5% of the elderly consumed fish two to three times a week, the rest less than two times.

### 4.2. Educational Status

After completing postsecondary education, there is a pivotal moment in a person’s life when they leave their home and move by themself or with their partner. This frequently coincides with a period of nutrition and lifestyle responsibilities, when people around 20–30 years old have little knowledge of grocery buying or cooking, as well as meal planning. The psychosocial, cultural, socioeconomic, and biological aspects are the determinants of food selection and intake [34]. A study published by Sui et al. evaluated the consumption patterns of different types of meats in Australian people. The authors described that the individuals with higher per-capita intakes especially ate chicken, while the people with the lowest per-capita intakes consumed game meat and organs [35]. Thus, our results were in accordance with the ones from the literature, chicken being eaten by young and middle-aged adults with a tertiary education.

Among the patients with HF who have graduated a maximum of 12 grades, there was a preference for meat, fruits, vegetables, potatoes, and bread. A less-balanced diet was observed in the case of patients in the control group, in which the favorite foods were salami and fast food. In the absence of a condition that makes them aware of the importance of healthy eating habits, they most likely prefer easy-to-obtain culinary products. In the case of those with higher education, potatoes and bread were in last place in culinary preferences, being overtaken by sweets. Moreover, only 13.3% of the subjects with high education preferred salami.

### 4.3. Employment Status

Previous studies showed that people who work overtime or night shifts are prone to a high level of stress and fatigue [36]. In addition, the availability of healthy food and social influence has been found to be positively associated with the overconsumption of unhealthy foods like salt, sugar, and fats. Similar associations occur in the case of people who have a sedentary job in the office but which involves an increased number of hours and a high level of responsibility. They tend to choose types of meat that cook easier, like chicken. Moreover, employees often fail to adopt a healthy lifestyle in their daily routine, and fast food is the solution that many turn to because they do not have time to cook or go out to lunch. So, they eat in a hurry inside the workplace. We noticed that non-full-time workers have a lower quality in the food they consumed, possibly in the context of their financial status.

### 4.4. Civil Status

Diversity is the dominant note in the eating habits of married patients. They consume both protein and dairy products, as well as vegetables, fruits, and sweets. A slightly more restrictive diet occurs in single patients, so sweets, bread, juices, and sour cream are missing. Previous studies have observed similar patterns in the general population. One explanation is that single people pay more attention to their diet in trying to maintain a normal body weight [37,38].

The lack of studies on patients with FH and similar food habits with the ones from our country makes it almost impossible to compare ours with other research. Even if these results are not published on FH patients, we can consider that eating any type of lean meat is important for patients diagnosed with FH, preferably grilled or boiled, in order to keep their LDL levels under control. 

## 5. Limitations of the Study

The limitations of this study are due to the low number of young patients, the use of a single nutrition questionnaire, and the lack of studies on the researched topic, which made it impossible to report the data of previous results. This last reason can also be seen as a positive aspect as a perfectible starting point.

## 6. Conclusions

Our study revealed that, although patients with FH avoid junk food, they still have a high intake of carbohydrates when compared to the control group. Overall, the patients included in our study had unhealthy eating habits: their daily meals lacked in fruits, vegetables, dairy products, and fish. We consider the implementation of educational nutritional programs should be mandatory even from primary school age, although this aspect is more important in the cases of patients with FH.

FH patients need a holistic approach. Awareness of the importance of a healthy lifestyle and compliance with medication recommendations is the responsibility of both physicians and patients. Given the complexity of the condition, and the fact that it is found in all age groups and involves long-term restrictions and limitations, management must be done with the help of a multidisciplinary team, consisting of an internist, cardiologist, nutritionist, and psychologist. A balanced approach, in which therapeutic success is doubled by a preserved quality of life, is ideal. Future studies that include supervised nutritional interventions are needed in order to obtain additional information on this as-yet unexplored topic.

## Figures and Tables

**Table 1:** General characteristics of the studied population.

General Characteristics		FH Group	Control Group	*p*-Value
Sex *n* (%)				
	Males	29 (41.4)	7 (35.0)	0.796
	Females	41 (58.6)	13 (65.0)	
Place of origin, *n* (%)				
	Rural	18 (25.7)	6 (30.0)	0.776
	Urban	52 (74.3)	14 (70.0)	
Age, years (mean ± SD)		54.65 ± 12.81	41.70 ± 13.51	<0.001
Education, *n* (%)				
	Gymnasium	7 (10)	2 (10.0)	1.000
	10 classes	11 (15.7)	1 (5.0)	0.287
	Highschool	23 (32.9)	2 (10.0)	0.051
	University	29 (41.4)	15 (75.0)	0.011
Employment status, *n* (%)				
	Full-time employed	36 (51.4)	16 (80.0)	0.038
	Part-time employed	3 (4.3)	1 (5.0)	1.000
	Retired	28 (40.0)	2 (10.0)	0.015
	Unemployed	3 (4.3)	1 (5.0)	1.000
Civil status, *n* (%)				
	Not married	2 (2.9)	6 (30.0)	0.001
	Married	56 (80.0)	12 (60.0)	0.081
	Divorced	5 (7.1)	1 (5.0)	1.000
	Widow/er	7 (10.0)	1 (5.0)	0.679
BMI, kg/m^2^ (mean ± SD)		28.75 ± 4.96	24.95 ± 6.19	0.005
Blood samples (mean ± SD/median (IQR))				
	Hemoglobin, mg/dL	13.60 ± 1.40	13.05 ± 1.42	0.121
	Uric acid, mg/dL	4.17 (3.43;5.06)	4.05 (3.44;4.91)	0.850
	Iron, µg/dL	95.46 ± 27.11	78.95 ± 24.37	0.016
	Urea, mg/dL	31.95 (26.17;39.32)	35.05 (32.82;36.57)	0.827
	Creatinine, mg/dL	0.99 (0.90;1.09)	0.94 (0.88;1.02)	0.246
	Clearance	72.68 ± 16.66	83.00 ±18.39	0.019
	Proteins, g/dL	7.18 (6.91;7.65)	7.47 (6.68;8.38)	0.139
	GOT, UI/L	19.85 (15.92;27.90)	17.35 (13.75;21.55)	0.113
	GPT, UI/L	20.20 (16.02;32.95)	17.50 (15.77;27.92)	0.455
	GGT, U/L	29.45 (19.40;57.82)	24.10 (20.00;24.10)	0.339
	Glucose, mg/dL	99.70 (90.20;99.70)	99.20 (92.47;103.95)	0.593
	HbA1c, %	5.46 (5.16;5.92)	5.22 (4.95;5.46)	0.028
	TC, mg/dL	278.00 (249.50;310.25)	201.00 (173.80;223.75)	<0.001
	HDL cholesterol, mg/dL	57.82 ± 16.52	46.56 ± 10.24	<0.001
	Non-HDL cholesterol, mg/dL	221.25 (186.45;253.42)	160.55 (132.30;176.60)	<0.001
	LDLc, mg/dL	196.02 ± 47.02	126.17 ± 40.40	<0.001
	Corrected LDL cholesterol, mg/dL	296.29 (224.84;389.75)	130.75 (97.10;155.87)	<0.001
	TG, mg/dL	164.70 (104.82;227.57)	100.30 (93.47;144.60)	0.007

GGT—gamma-glutamyl transpeptidase; GOT—aspartate transaminase; GPT—alanine aminotransferase; HbA1c—glycated hemoglobin; HDL—high-density lipoprotein; IQR—interquartile range; LDL—low-density lipoprotein; LDLc—corrected low-density lipoprotein; SD—standard deviation; TC—total cholesterol; TG—triglycerides; FH—familial hypercholesterolemia; BMI—body mass index.

**Table 2:** Dutch Lipid Clinic Network diagnostic criteria applied to the studied population.

DLCN Criteria	FH	Controls	*p*-Value
First-degree relative			
Premature CVD	33 (47.1%)	2 (10.0%)	0.043
LDL > 95th percentile	21 (30.0%)	3 (15.0%)	0.255
Tendinous xanthoma a/o arcus cornealis	11 (15.7%)	1 (5.0%)	0.287
Child with LDL > 95th percentile	23 (32.9%)	1 (5.0%)	0.019
Personal history			
Premature CVA/PVD	8 (11.4%)	0 (0%)	-
Coronary artery disease	25 (35.7%)	3 (15.0%)	0.102
Physical examination			
Arcus cornealis < 45 years of age	33 (47.1%)	1 (5.0%)	<0.001
LDL			
155–189 mg/dL	0 (0%)	7 (35.0%)	-
190–249 mg/dL	24 (34.3%)	0 (0%)	-
250–329 mg/dL	18 (25.7%)	0 (0%)	-
>330 mg/dL	28 (40.0%)	0 (0%)	-

FH—familial hypercholesterolemia; CVD—cardiovascular disease; LDL—low-density lipoprotein; CVA—cerebrovascular accident; PVD—peripheral vascular disease.

**Table 3:** Favorite foods.

Favorite Food	FH, *n* (%)	Control, *n* (%)	*p*-Value
Bread	41 (58.6)	11 (55.0)	0.802
Meat	54 (77.1)	12 (60.0)	0.155
Vegetables	59 (84.3)	15 (75.0)	0.337
Cakes	35 (50.0)	6 (30.0)	0.133
Cheese	53 (75.7)	12 (60.0)	0.256
Dairy products	47 (67.1)	11 (55.0)	0.427
Juices	10 (14.3)	6 (30.0)	0.180
Sweets	34 (48.6)	11 (55.0)	0.800
Fruits	54 (77.1)	14 (70.0)	0.560
Sour cream	30 (42.9)	2 (10.0)	0.007
Potatoes	46 (65.7)	11 (55.0)	0.435
Salami	25 (35.7)	5 (25.0)	0.431
Fast food	13 (18.6)	7 (35.0)	0.135

**Table 4:** Fats used for cooking or consumed in the two studied groups.

Types of Fats Used	FH, *n* (%)	Control, *n* (%)	*p*-Value
Butter	34 (48.6)	14 (70.0)	0.128
Lard	11 (15.7)	3 (15.0)	1.000
Margarine	13 (18.6)	0 (0.0)	-
Oil	63 (90.0)	19 (95.0)	0.679
Sour cream	32 (45.7)	3 (15.0)	0.018
Bacon	15 (21.4)	2 (10.0)	0.342
Whipped cream	11 (15.7)	1 (5.0)	0.287

**Table 5:** Frequency of the eating habits.

Favorite Food	FH, Median (IQR)	Control, Median (IQR)	*p*-Value
Bread	4 (3;4)	4 (2.25;4)	0.334
Pasta	1 (1;2)	2 (1;2)	0.154
Potatoes	2 (1;2)	2 (2;2)	0.813
Green peas	1 (1;2)	1 (1;1)	0.035
Other vegetables	3 (2;4)	3 (2;3)	0.518
Fruits	3 (2.75;4)	3 (2;3)	0.172
Dairy products	2 (2;3)	2 (2;3)	0.655
Beef/pork	2 (1;3)	2 (1;2)	0.481
Chicken	2 (1;3)	2 (1;2.75)	0.880
Fish	1 (1;2)	1.5 (1;2)	0.281
Fried food	2 (1;3)	2 (2;3)	0.162
Sweets	2 (1;3)	2.5 (2;3)	0.216
Soft juices	1 (1;1)	1 (1;1)	0.012
Fast food	1 (1;1)	1 (1;1)	0.815

1—less than 2 times/week, 2—2–3 times/week, 3—4–6 times/week, and 4—daily.

**Table 6:** What situation makes you eat more?

	FH, *n* (%)	Control, *n* (%)	*p*-Value
Loneliness	14 (20)	0 (0)	-
Boredom	29 (41.4)	4 (20.0)	0.114
Sadness	23 (32.9)	2 (10.0)	0.051
Depression	10 (14.3)	0 (0)	-
Stress	24 (34.3)	11 (55.0)	0.121
In society	15 (21.4)	9 (45.0)	0.047
Weekend	29 (41.4)	8 (40.0)	1.000
Television	18 (25.7)	4 (20.0)	0.771

**Table 7:** What do you feel after you eat more than you should?

	FH, *n* (%)	Control, *n* (%)	*p*-Value
Depression	7 (10.0)	0 (0)	-
Guilt	22 (31.4)	4 (20.0)	0.409
Satisfaction	9 (12.9)	3 (15.0)	0.725
Nothing	19 (27.1)	7 (35.0)	0.578
Skip next meal	18 (25.7)	8 (40.0)	0.265
Exercise	21 (30.0)	3 (15.0)	0.255

## Data Availability

Not applicable.

## Supplementary Materials

The following supporting information can be downloaded at https://www.mdpi.com/article/10.3390/nu14153124/s1: Table S1: Favorite food and statistically significant correlations; Table S2: Favorite food; Table S3: Types of fats used for cooking or consumed and statistically significant correlations; Table S4: Fats used for cooking or consumed; Table S5: Frequency of the eating habits and statistically significant differences; Table S6: Frequency of the eating habits; Table S7: What situation makes you eat more?; Table S8: What do you feel after you eat more than you should?
