# Supplementary material for: Eating Habits in Patients with Familial Hypercholesterolemia from North-Eastern Romania
Source: Nutrients. 2022 Jul 29;14(15):3124. doi: 10.3390/nu14153124 (PMC9370791; doi:10.3390/nu14153124)
Supplement: Supplementary file 1 [file nutrients-14-03124-s001.zip › nutrients-1806203-supplementary.pdf]

Table S1. Favorite food and statistically significant correlations.

Favorite food	Yes (mean ± SD / median (IQR))	No (mean ± SD / median (IQR))	*p*-value
**FH group**			
**Meat**			
Hemoglobin	13.80 (12.70; 14.95)	13.00 (12.45; 13.75)	0.013
**Salami**			
BMI	30.80 ± 4.84	27.61 ± 4.71	0.009
Hemoglobin	14.30 (13.10; 15.15)	13.10 (12.45; 14.20)	0.015
Glucose	109.20 (93.45; 131.93)	97.20 (89.35; 106.35)	0.033
HbA1c	5.84 (5.32; 6.46)	5.43 (5.10; 5.76)	0.014
GPT	29.30 (17.35; 51.80)	18.00 (15.10; 30.1)	0.012
**Fruits**			
HDL-cholesterol	60.83 ± 15.94	47.64 ± 14.62	0.004
**Bread**			
BMI	29.79 ± 5.09	27.28 ± 4.53	0.036
GGT	38.10 (21.05; 100.30)	23.00 (19.15; 42.53)	0.015
**Sour cream**			
GOT	24.70 (19.02; 31.05)	17.65 (15.00; 22.52)	0.007
GPT	28.80 (17.50; 49.50)	17.60 (15.72; 28.82)	0.019
GGT	44.60 (25.80; 105.55)	22.60 (18.07; 41.70)	<0.001
**Potatoes**			
BMI	29.75 ± 5.06	26.83 ± 4.24	0.018
HbA1c	5.76 (5.21; 6.03)	5.35 (4.95; 5.70)	0.020
GGT	38.55 (24.35; 85.10)	19.95 (17.82; 37.32)	0.001
**Cakes**			
Creatinine	0.92 (0.86; 1.08)	1.04 (0.93; 1.13)	0.040
**Sweets**			
Glucose	102.70 (91.30; 114.80)	98.20 (88.10; 109.20)	0.046
**Fast food**			
GPT	28.30 (18.95; 62.70)	18.90 (15.50; 31.75)	0.043
**Control group**			
**Vegetables**			
Hemoglobin	13.44 ± 0.96	11.86 ± 1.99	0.026
LDL-cholesterol	140.76 ± 31.17	82.42 ± 34.06	0.002
**Cheese**			
Hemoglobin	13.81 ± 0.75	11.90 ± 1.44	0.001
Proteins	8.09 (7.38; 8.70)	6.70 (6.66; 7.53)	0.016
Creatinine clearance	89.91 ± 15.42	72.62 ± 18.42	0.035
**Dairy products**			
Iron	88.68 ± 27.00	67.05 ± 14.50	0.037
GPT	24.10 (19.01; 31.40)	15.70 (12.40; 20.35)	0.046
**Cakes**			
GGT	18.30 (14.47; 27.17)	28.10 (22.02; 55.25)	0.041
**Juices**			
Triglycerides	92.25 (75.65; 99.22)	117.80 (94.52; 170.15)	0.033

FH, familial hypercholesterolemia; BMI, body mass index; HbA1c, glycated hemoglobin; GPT, alanine aminotransferase; HDL, high-density lipoprotein; GGT, gamma-glutamyl transferase; GOT, aspartate transaminase; LDL, low-density lipoprotein; SD, standard deviation; IQR, Inter-quartile range.

Table S2. Favorite food.

	18–24 years	25–34 years	35–44 years	45–54 years	55–64 years	65–74 years	>75 years
Bread	0 (0%)	2 (66.7%)	3 (42.9%)	13 (65%)	12 (54.5%)	9 (75%)	2 (50%)
Meat	2 (100%)	2 (66.7%)	6 (85.7%)	16 (80%)	17 (77.3%)	8 (66.7%)	3 (75%)
Vegetables	2 (100%)	1 (33.3%)	7 (100%)	16 (80%)	21 (95.5%)	8 (66.7%)	4 (100%)
Cakes	1 (50%)	3 (100%)	4 (57.1%)	12 (60%)	9 (40.9%)	4 (33.3%)	2 (50%)
Cheese	0 (0%)	2 (66.7%)	7 (100%)	12 (60%)	18 (81.8%)	11 (91.7%)	3 (75%)
Dairy products	1 (50%)	1 (33.3%)	5 (71.4%)	11 (55%)	17 (77.3%)	9 (75%)	3 (75%)
Juices	0 (0%)	0 (0%)	3 (42.9%)	3 (15%)	2 (9.1%)	1 (8.3%)	1 (25%)
Sweets	0 (0%)	3 (100%)	5 (71.4%)	13 (65%)	9 (40.9%)	3 (25%)	1 (25%)
Fruits	1 (50%)	2 (66.7%)	5 (71.4%)	16 (80%)	20 (90.9%)	7 (58.3%)	3 (75%)
Sour cream	0 (0%)	1 (33.3%)	3 (42.9%)	8 (40%)	13 (59.1%)	4 (33.3%)	1 (25%)
Potatoes	1 (50%)	2 (66.7%)	3 (42.9%)	15 (75%)	13 (59.1%)	9 (75%)	3 (75%)
Salami	1 (50%)	1 (33.3%)	2 (28.6%)	7 (35%)	9 (40.9%)	4 (33.3%)	1 (25%)
Fast-food	1 (50%)	1 (33.3%)	2 (28.6%)	4 (20%)	3 (13.6%)	2 (16.7%)	0 (0%)

Table S3. Types of fats used for cooking or consumed and statistically significant correlations.

Preferred fats	Yes (mean ± SD / median (IQR))	No (mean ± SD / median (IQR))	*p*-value
**Bacon**			
Hemoglobin	14.54 ± 1.08	13.35 ± 1.38	0.003
GGT	47.60 (32.00; 98.60)	25.80 (18.80; 46.00)	0.008
GPT	31.00 (17.50; 49.80)	18.90 (15.30; 31.30)	0.022
Glucose	108.50 (97.20; 115.40)	96.90 (88.50; 108.10)	0.045
Glycated hemoglobin	5.88 (5.43; 6.13)	5.42 (5.09; 5.82)	0.014
Uric acid	4.95 (4.28; 5.38)	3.86 (3.36; 4.77)	0.008
Tryglicerides	217.30 (129.30; 377.70)	157.00 (103.10; 205.00)	0.019
**Butter**			
Iron	88.51 ± 24.49	102.02 ± 28.15	0.036
GGT	25.20 (18,55; 43.62)	40.50 (22.10; 80.35)	0.042
Urea	30.90 (25.22; 30.90)	36.90 (28.87; 40.67)	0.022
Glucose	96.10 (86.62; 96.10)	103.15 (91.90; 114.60)	0.042
Glycated hemoglobin	5.33 (5.01; 5.83)	5.59 (5.30; 6.00)	0.049
**Sour cream**			
GGT	44.60 (23.7; 103.85)	24.70 (19.02; 39.30)	0.003
GOT	24.1 (17.9; 30.95)	17.40 (14.87; 23.07)	0.007
GPT	29.70 (18.00; 48.30)	17.10 (14.65; 25.45)	0.001
Proteins	7.33 (7.05; 7.78)	7.11 (6.83; 7.58)	0.036
Uric acid	4.55 (3.60; 5.26)	3.84 (3.27; 4.66)	0.038
**Oil**			
Creatinine	1.02 (0.91; 1.12)	0.087 (0.076; 0.095)	0.009
Uric acid	4.32 (3.49; 5.17)	3.42 (2.89; 3.90)	0.021
Triglycerides	167.00 (117.79; 235.90)	86.50 (67.50; 120.50)	0.029
**Margarine**			
Proteins	7.62 (7.12; 7.83)	7.14 (6.87; 7.43)	0.017

Table S4. Fats used for cooking or consumed.

	18–24 years	25–34 years	35–44 years	45–54 years	55–64 years	65–74 years	>75 years
Butter	2 (100%)	3 (100%)	3 (42.9%)	8 (40%)	11 (50%)	5 (41.7%)	2 (50%)
Lard	0 (0%)	0 (0%)	0 (0%)	5 (25%)	1 (4.5%)	4 (33.3%)	1 (25%)
Margarine	0 (0%)	0 (0%)	1 (14.3%)	6 (30%)	3 (13.6%)	2 (16.7%)	1 (25%)
Oil	1 (50%)	3 (100%)	6 (85.7%)	18 (90%)	21 (95.5%)	11 (91.7%)	3 (75%)
Sour cream	0 (0%)	3 (100%)	4 (57.1%)	9 (45%)	12 (54.5%)	4 (33.3%)	0 (0%)
Bacon	0 (0%)	0 (0%)	0 (0%)	7 (35%)	5 (22.7%)	1 (8.3%)	2 (50%)
Whipped cream	0 (0%)	0 (0%)	1 (14.3%)	5 (25%)	3 (13.6%)	2 (16.7%)	0 (0%)

Table S5. Frequency of the eating habits and statistically significant differences.

**FH group**			***p*-value**
Other vegetables			
Urea	2<br>27.25 (21.30; 31.39)	3<br>36.90 (29.90; 40.20)	0.029
Beef/pork			
Urea	1<br>31.1 (23.30; 33.65)	3<br>38.15 (30.47; 42.45)	0.031
Fish			
GOT	1<br>20.50 (16.57; 27.97)	3<br>12.70 (11.62; 14.07)	0.009
	2<br>20.30 (16.60; 28.05)	3<br>12.70 (11.62; 14.07)	0.013
GPT	1<br>20.55 (16.12; 36.52)	3<br>10.85 (8.22; 15.65)	0.026
	2<br>21.55 (16.57; 33.85)	3<br>10.85 (8.22; 15.65)	0.021
TC	2<br>296.00 (257.00; 331.50)	3<br>210 (145.75; 261.50)	0.025
Non-HDL	2<br>247.55 (199.80; 267.30)	3<br>140.95 (98.92; 188.82)	0.009

1—less than 2 times/week, 2—2–3 times/week, 3—4–6 times/week, 4—daily. TC, total cholesterol.

Table S6. Frequency of the eating habits.

	18–24 years	25–34 years	35–44 years	45–54 years	55–64 years	65–74 years	>75 years
**Bread**							
<2x/week	0 (0%)	0 (0%)	2 (28.6%)	0 (0%)	1 (4.5%)	0 (0%)	0 (0%)
2–3x/week	2 (100%)	0 (0%)	0 (0%)	2 (10%)	4 (18.2%)	1 (8.3%)	0 (0%)
4–6x/week	0 (0%)	1 (33.3%)	1 (14.3%)	2 (10%)	1 (4.5%)	5 (41.7%)	2 (50%)
Daily	0 (0%)	2 (66.7%)	4 (57.1%)	16 (72.7%)	16 (72.7%)	6 (50%)	2 (50%)
**Pasta**							
<2x/week	2 (100%)	2 (66.7%)	3 (42.9%)	11 (55.5%)	16 (72.7%)	6 (50%)	2 (50%)
2–3x/week	0 (0%)	0 (0%)	4 (57.1%)	7 (35%)	5 (22.7%)	6 (50%)	2 (50%)
4–6x/week	0 (0%)	0 (0%)	0 (0%)	1 (5%)	1 (4.5%)	0 (0%)	0 (0%)
Daily	0 (0%)	1 (33.3%)	0 (0%)	1 (5%)	0 (0%)	0 (0%)	0 (0%)
**Potatoes**							
<2x/week	1 (50%)	1 (33.3%)	2 (28.6%)	4 (20%)	12 (54.5%)	2 (16.7%)	0 (0%)
2–3x/week	0 (0%)	2 (66.7%)	4 (57.1%)	12 (60%)	7 (31.8%)	7 (58.3%)	3 (75%)
4–6x/week	1 (50%)	0 (0%)	1 (14.3%)	3 (15%)	3 (13.6%)	2 (16.7%)	1 (25%)
Daily	0 (0%)	0 (0%)	0 (0%)	1 (5%)	0 (0%)	1 (8.3%)	0 (0%)
**Green peas**							
<2x/week	1 (50%)	3 (100%)	5 (71.4%)	11 (55%)	14 (63.6%)	6 (50%)	2 (50%)
2–3x/week	1 (50%)	0 (0%)	2 (28.6%)	8 (40%)	8 (36.4%)	4 (33.3%)	1 (25%)
4–6x/week	0 (0%)	0 (0%)	0 (0%)	1 (5%)	0 (0%)	2 (16.7%)	1 (25%)
Daily	0 (0%)	0 (0%)	0 (0%)	0 (0%)	0 (0%)	0 (0%)	0 (0%)
**Other vegetables**							
<2x/week	0 (0%)	0 (0%)	0 (0%)	4 (20%)	2 (9.1%)	1 (8.3%%)	1 (25%)
2–3x/week	0 (0%)	2 (66.7%)	0 (0%)	6 (30%)	4 (18.2%)	1 (8.3%)	1 (25%)
4–6x/week	1 (50%)	1 (33.3%)	2 (28.6%)	7 (35%)	9 (40.9%)	7 (58.3%)	0 (0%)
Daily	1 (50%)	0 (0%)	5 (71.4%)	3 (15%)	7 (31.8%)	3 (25%)	2 (50%)
**Fruits**							
<2x/week	1 (50%)	0 (0%)	0 (0%)	3 (15%)	1 (4.5%)	2 (16.7%)	0 (0%)
2–3x/week	0 (0%)	0 (0%)	1 (14.3%)	4 (20%)	3 (13.6%)	2 (16.7%)	0 (0%)
4–6x/week	0 (0%)	3 (100%)	1 (14.3%)	8 (40%)	6 (27.3%)	5 (41.7%)	1 (25%)
Daily	1 (50%)	0 (0%)	5 (71.4%)	5 (25%)	12 (54.5%)	3 (25%)	3 (75%)
**Dairy products**							
<2x/week	1 (50%)	0 (0%)	0 (0%)	7 (35%)	4 (18.2%)	0 (0%)	1 (25%)
2–3x/week	1 (50%)	1 (33.3%)	1 (14.3%)	7 (35%)	10 (45.5%)	7 (58.3%)	1 (25%)
4–6x/week	0 (0%)	2 (66.7%)	2 (28.6%)	4 (20%)	6 (27.3%)	2 (16.7%)	1 (25%)
Daily	0 (0%)	0 (0%)	4 (57.1%)	2 (10%)	2 (9.1%)	3 (25%)	1 (25%)
**Beef/pork**							
<2x/week	1 (50%)	1 (33.3%)	0 (0%)	6 (30%)	9 (40.9%)	3 (25%)	1 (25%)
2–3x/week	0 (0%)	1 (33.3%)	5 (71.4%)	9 (45%)	7 (31.8%)	5 (41.7%)	1 (25%)
4–6x/week	1 (50%)	1 (33.3%)	2 (28.6%)	4 (20%)	6 (27.3%)	4 (33.3%)	2 (50%)
Daily	0 (0%)	0 (0%)	0 (0%)	1 (5%)	0 (0%)	0 (0%)	0 (0%)
**Chicken**							
<2x/week	0 (0%)	0 (0%)	1 (14.3%)	2 (10%)	7 (31.8%)	6 (50%)	4 (100%)
2–3x/week	1 (50%)	1 (33.3%)	2 (28.6%)	14 (70%)	10 (45.5%)	4 (33.3%)	0 (0%)
4–6x/week	0 (0%)	2 (66.7%)	3 (42.9%)	2 (10%)	4 (18.2%)	2 (16.7%)	0 (0%)
Daily	1 (50%)	0 (0%)	1 (14.3%)	2 (10%)	1 (4.5%)	0 (0%)	0 (0%)
**Fish**							

<2x/week	1 (50%)	3 (100%)	3 (42.9%)	14 (70%)	13 (59.1%)	7 (58.3%)	3 (75%)
2–3x/week	1 (50%)	0 (0%)	2 (28.6%)	4 (20%)	9 (40.9%)	5 (41.7%)	1 (25%)
4–6x/week	0 (0%)	0 (0%)	2 (28.6%)	2 (10%)	0 (0%)	0 (0%)	0 (0%)
Daily	0 (0%)	0 (0%)	0 (0%)	0 (0%)	0 (0%)	0 (0%)	0 (0%)
**Fried food**							
<2x/week	0 (0%)	1 (33.3%)	3 (42.9%)	4 (20%)	8 (36.4%)	3 (25%)	2 (50%)
2–3x/week	1 (50%)	1 (33.3%)	2 (28.6%)	8 (40%)	7 (31.8%)	4 (33.3%)	1 (25%)
4–6x/week	1 (50%)	1 (33.3%)	2 (28.6%)	7 (35%)	6 (27.3%)	4 (33.3%)	1 (25%)
Daily	0 (0%)	0 (0%)	0 (0%)	1 (5%)	1 (4.5%)	1 (8.3%)	0 (0%)
**Sweets**							
<2x/week	0 (0%)	0 (0%)	1 (14.3%)	4 (20%)	7 (31.8%)	6 (50%)	2 (50%)
2–3x/week	2 (100%)	0 (0%)	3 (42.9%)	9 (45%)	8 (36.4%)	5 (41.7%)	1 (25%)
4–6x/week	0 (0%)	2 (66.7%)	1 (14.3%)	6 (30%)	4 (18.2%)	0 (0%)	0 (0%)
Daily	0 (0%)	1 (33.3%)	2 (28.6%)	1 (5%)	3 (13.6%)	1 (8.3%)	1 (25%)
**Soft juices**							
<2x/week	2 (100%)	3 (100%)	3 (42.9%)	16 (80%)	18 (81.8%)	11 (91.7%)	4 (100%)
2–3x/week	0 (0%)	0 (0%)	3 (42.9%)	2 (10%)	3 (13.6%)	1 (8.3%)	0 (0%)
4–6x/week	0 (0%)	0 (0%)	0 (0%)	2 (10%)	1 (4.5%)	0 (0%)	0 (0%)
Daily	0 (0%)	0 (0%)	1 (14.3%)	0 (0%)	0 (0%)	0 (0%)	0 (0%)
**Fast-food**							
<2x/week	1 (50%)	2 (66.7%)	4 (57.1%)	16 (80%)	21 (95.5%)	10 (83.3%)	4 (100%)
2–3x/week	1 (50%)	0 (0%)	2 (28.6%)	4 (20%)	1 (4.5%)	2 (16.7%)	0 (0%)
4–6x/week	0 (0%)	1 (33.3%)	0 (0%)	0 (0%)	0 (0%)	0 (0%)	0 (0%)
Daily	0 (0%)	0 (0%)	1 (14.3%)	0 (0%)	0 (0%)	0 (0%)	0 (0%)

Table S7. What situation makes you eat more?

	**18–24 years**	**25–34 years**	**35–44 years**	**45–54 years**	**55–64 years**	**65–74 years**	**>75 years**
Loneliness	0 (0%)	0 (0%)	0 (0%)	6 (30%)	5 (22.7%)	2 (16.7%)	1 (25%)
Boredom	0 (0%)	1 (33.3%)	3 (42.9%)	10 (50%)	10 (45.5%)	5 (41.7%)	0 (0%)
Sadness	0 (0%)	1 (33.3%)	2 (28.6%)	8 (40%)	6 (27.3%)	4 (33.3%)	2 (50%)
Depression	0 (0%)	0 (0%)	1 (14.3%)	3 (15%)	3 (13.6%)	2 (16.7%)	1 (25%)
Stress	1 (50%)	2 (66.7%)	3 (42.9%)	8 (40%)	6 (27.3%)	3 (25%)	1 (25%)
In society	0 (0%)	0 (0%)	2 (28.6%)	4 (20%)	3 (13.6%)	4 (33.3%)	2 (50%)
Weekend	1 (50%)	1 (33.3%)	4 (57.1%)	8 (40%)	7 (31.8%)	5 (41.7%)	3 (75%)
Television	0 (0%)	2 (66.7%)	0 (0%)	7 (35%)	5 (22.7%)	3 (25%)	1 (25%)

Table S8. What do you feel after you eat more than you should?

	**18–24 years**	**25–34 years**	**35–44 years**	**45–54 years**	**55–64 years**	**65–74 years**	**>75 years**
Depression	0 (0%)	0 (0%)	0 (0%)	2 (10%)	4 (18.2%)	1 (8.3%)	0 (0%)
Guilt	0 (0%)	2 (66.7%)	2 (28.6%)	7 (35%)	8 (36.4%)	3 (25%)	0 (0%)
Satisfaction	0 (0%)	2 (66.7%)	0 (0%)	2 (10%)	3 (13.6%)	2 (16.7%)	0 (0%)
Nothing	2 (100%)	0 (0%)	2 (28.6%)	5 (25%)	6 (27.3%)	0 (0%)	4 (100%)
Skip next meal	0 (0%)	2 (66.7%)	3 (42.9%)	4 (20%)	3 (13.6%)	5 (41.7%)	1 (25%)
Exercise	0 (0%)	0 (0%)	2 (28.6%)	7 (35%)	6 (27.3%)	6 (50%)	0 (0%)
